# Nicotianamine, a Novel Enhancer of Rice Iron Bioavailability to Humans

**DOI:** 10.1371/journal.pone.0010190

**Published:** 2010-04-16

**Authors:** Luqing Zheng, Zhiqiang Cheng, Chunxiang Ai, Xinhang Jiang, Xiaoshu Bei, Ye Zheng, Raymond P. Glahn, Ross M. Welch, Dennis D. Miller, Xin Gen Lei, Huixia Shou

**Affiliations:** 1 State Key Laboratory of Plant Physiology and Biochemistry, College of Life Sciences, Zhejiang University, Hangzhou, China; 2 Departments of Animal Science and Food Science, Cornell University, Ithaca, New York, United States of America; 3 United State Department of Agriculture-Agricultural Research Service, Robert W. Holley Center for Agriculture and Health, Cornell University, Ithaca, New York, United States of America; University of Melbourne, Australia

## Abstract

**Background:**

Polished rice is a staple food for over 50% of the world's population, but contains little bioavailable iron (Fe) to meet human needs. Thus, biofortifying the rice grain with novel promoters or enhancers of Fe utilization would be one of the most effective strategies to prevent the high prevalence of Fe deficiency and iron deficiency anemia in the developing world.

**Methodology/Principal Findings:**

We transformed an elite rice line cultivated in Southern China with the rice nicotianamine synthase gene (*OsNAS1*) fused to a rice glutelin promoter. Endosperm overexpression of *OsNAS1* resulted in a significant increase in nicotianamine (NA) concentrations in both unpolished and polished grain. Bioavailability of Fe from the high NA grain, as measured by ferritin synthesis in an *in vitro* Caco-2 cell model that simulates the human digestive system, was twice as much as that of the control line. When added at 1∶1 molar ratio to ferrous Fe in the cell system, NA was twice as effective when compared to ascorbic acid (one of the most potent known enhancers of Fe bioavailability) in promoting more ferritin synthesis.

**Conclusions:**

Our data demonstrated that NA is a novel and effective promoter of iron utilization. Biofortifying polished rice with this compound has great potential in combating global human iron deficiency in people dependent on rice for their sustenance.

## Introduction

Iron (Fe) deficiency is the most prevalent nutrient deficiency in the world afflicting over 50% of the world population[1], [2]. Inadequate intake of iron and consumption of foods low in bioavailable iron are the major causes of this problem. Compared with heme-iron derived from animal foods, non-heme iron, the major form of iron in plant foods, is much less bioavailable (2 to 10%) from the diet[3], [4]. The low bioavailability of non-heme iron in these foods is attributed to the high amounts of inhibitors of iron absorption (i.e., phytate and polyphenolics)[5]. Although promoter compounds of iron utilization such as ascorbic acid (AA)[6], [7] and ethylenediaminetetraacetic acid (EDTA)[8] have been used as dietary fortificants to improve human iron nutritional status[6], this approach has limited accessibility or sustainability to resource-poor people afflicted with iron deficiency in the Global South. Alternatively, biofortifying staple crops with enhancers of iron absorption would be a more effective and sustainable solution. However, past efforts have focused mainly on increasing the total iron concentration in edible portions of food crops[9], [10], [11]. Little effort or progress has been made in exploring new plant compounds that promote bioavailability of iron from food staples.

Nicotianamine (NA) is biosynthesized from three molecules of *S*-adenosylmethionine (SAM) by NA synthase (NAS)[12], [13]. As a transition metal-chelator, NA facilitates the intra- and intercellular transport of essential trace metal cations, including Fe^2+^, Fe^3+^ and Zn^2+^, in plants[14]. Ectopic expression of the *Arabidopsis NAS* gene in tobacco resulted in a six-fold increase in NA level and a significant increase of Fe, Zn and manganese concentrations in leaves of adult plants[15]. A recent study showed that activation of *OsNAS3* led to increase of Fe, Zn in both green tissue and mature seed. Anemic mice fed with the *OsNAS3* activated transgenic rice seeds recovered to normal levels of hemoglobin and hematocrit within 2 weeks[16]. Because of these positive effects of NA on iron uptake and accumulation in plant roots and seeds[15], [17], we postulated that elevating NA in the edible portions of rice grain might improve iron bioavailability to animal or humans by chelating iron to form a soluble NA-ferrous complex. Therefore, we over expressed the *OsNAS1* gene in rice-grain endosperm, and obtained a significant increase of NA concentrations in the polished rice. Using a well-characterized *in vitro* Caco-2 cell (human epithelial colorectal adenocarcinoma cells) model for predicting bioavailability of iron in food[18], [19], we demonstrate that the polished rice from the *OsNAS1* transgenic lines displayed twice as much bioavailable iron as that of the non-transgenic control line. Responses of ferritin synthesis in the Caco-2 cells to the addition of NA in rice digests or ferrous sulfate solutions revealed that NA is a more potent promoter than AA, the strongest promoter of iron utilization currently identified. Overall, our findings indicate a great potential for biofortifying rice with NA to help eradicate iron deficiency in populations consuming rice as their staple food.

## Results

### Overexpressing *OsNAS1* in rice endosperm

**Figure 1:**
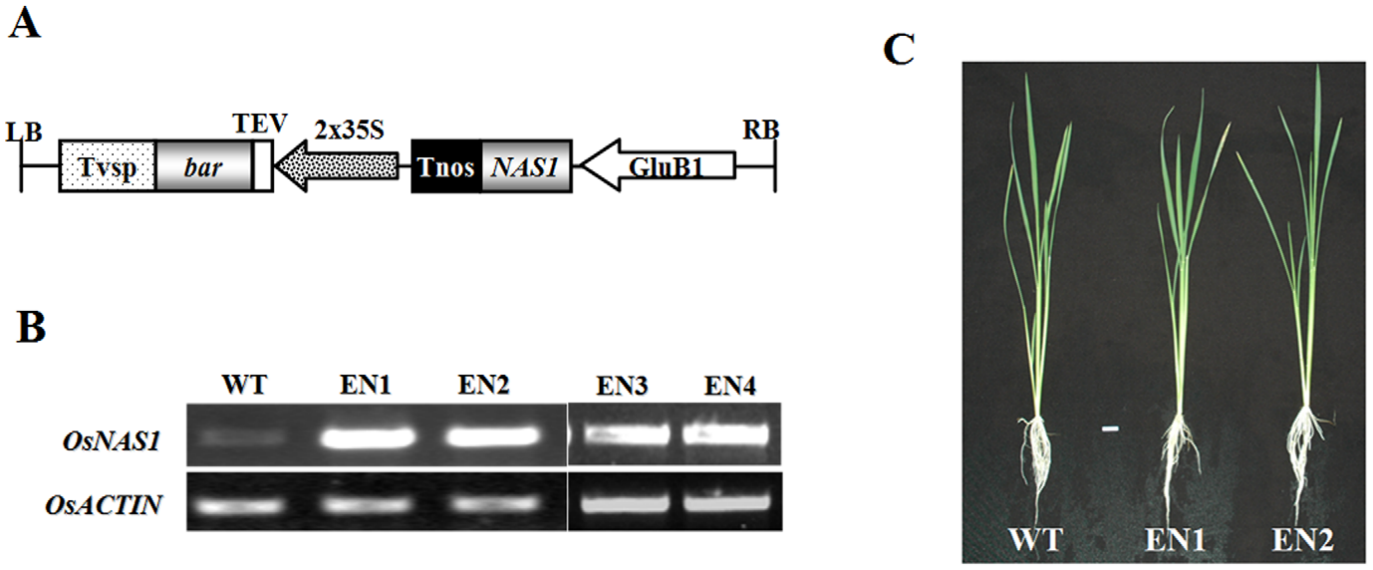
Overexpression of the *OsNAS1* gene in rice endosperm. (A) Schematic representation of the T-DNA in rice transformation vector. GluB-1, 2.3 kilo-base-pair rice glutelin B1 promoter; *NAS1*, coding sequence of rice *OsNAS1* gene; Tnos, nopaline synthase terminator; 2×35S, double CaMV 35S promoter; TEV, tobacco etch virus 5′ untranslated region; *bar*: phosphinothricin acetyltransferase gene; Tvsp: soybean vegetative storage protein terminator; LB and RB, left and right T-DNA borders, respectively. (B) Reverse transcriptase PCR of *OsNAS1* in EN1-EN4 transgenic and WT immature grains. EN1-EN4 are four transgenic lines overexpressing *OsNAS1* in endosperm. *OsACTIN* was used as the internal standard. Total RNAs were extracted from the immature seeds 18 days after pollination. (C) Thirty five day-old seedlings of EN1, EN2, and WT grown in nutrient solutions, bar = 2 cm.

The 2.3 kb promoter region of rice glutelin B1 gene (GluB-1, accession number AY427569)[20] was used to drive the rice NA synthase gene (*OsNAS1*, accession number AB021746) expression in rice endosperm. In addition, the T-DNA region of the binary vector (used for rice transformation) contained a selectable marker gene *bar* for the herbicide bialaphos resistance (Fig. 1A). The elite japonica rice variety, Xiushui 110 (wild type, WT), was used as the recipient of *Agrobacterium*-mediated transformation. Integration of the *OsNAS1* in seven independent transgenic lines, designated as EN1 to EN7, was confirmed by PCR and Southern blot analysis (data not shown). Reverse transcriptase PCR (RT-PCR) analysis was performed using RNA samples extracted from T_2_ immature seeds of four independent transgenic lines EN1 to EN4 to verify the expression of *OsNAS1* in the endosperms of transgenic seeds. The endosperm expression of *OsNAS1* resulted in a substantial increase of the *NAS1* transcript in EN1 to EN4 seeds over control seeds (Fig. 1B). The overexpression of *OsNAS1* in seed showed no obvious effect on agronomic traits, including the duration of maturation, plant height, tiller number, and seed weight evaluated in both field (Table 1) and hydroponic experiments (Fig. 1C).

**Table 1:** Agronomic performance of transgenic line EN1, EN2 and the wild type.

Genotypes	Duration of maturation (days)	Plant height (cm)	Tiller numbers	Grain no. per main panicle	1000-grain weight (g)	Grain yield per plant (g)
**WT**	157	81.7±4.0a	18.7±1.0a	141.7±21.4a	24.4±0.1a	33.5±1.3a
**EN1**	157	80.3±3.2a	19.1±1.3a	122.0±28.2a	24.4±0.1a	31.5±4.2a
**EN2**	157	82.2±2.0a	19.5±1.5a	136.3±23.5a	24.2±0.2a	34.5±2.7a

**Notes:** All values represent means ± SD of 10 plants of each line; Means with different letters in same column are significantly different (P<0.05, LSD test). WT, wild type; EN1 and EN2, Glu1-NAS1 transgenic line 1 and 2.

### Overexpressing *OsNAS1* leads to elevated NA concentrations in transgenic rice seed

**Figure 2:**
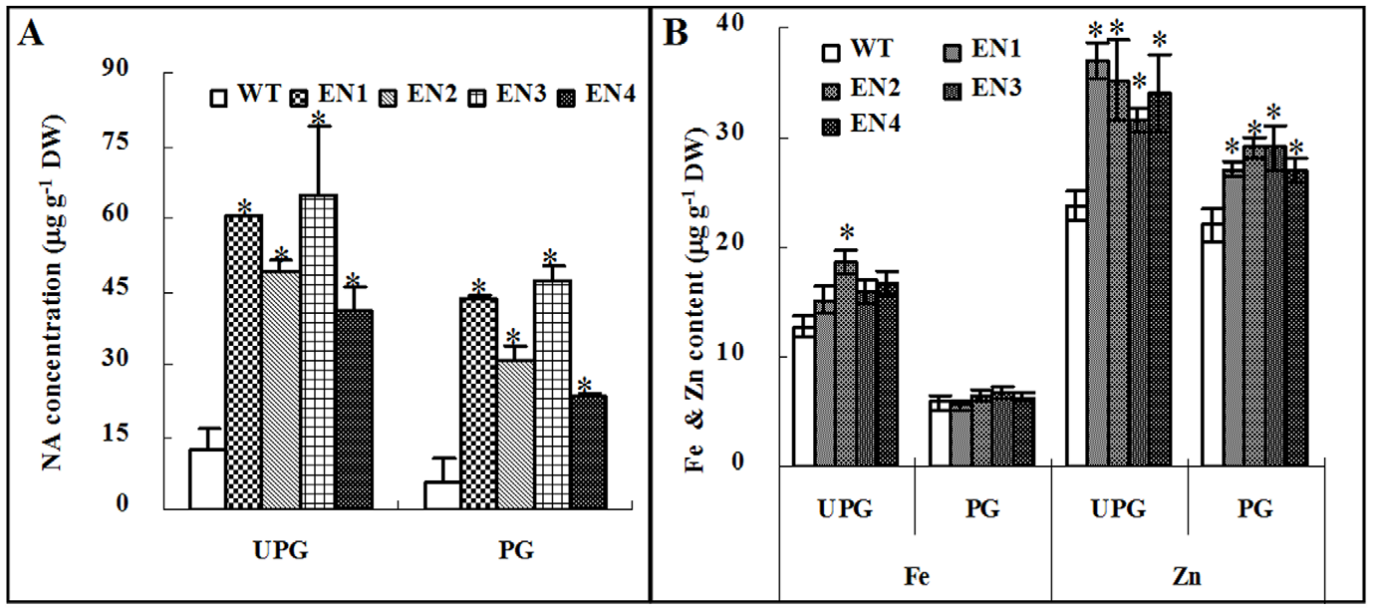
Nicotianamine (NA), Fe and Zn concentrations in the wild type (WT) and transgenic rice grain (EN1, EN2, EN3 and EN4). (A) NA concentration in unpolished grain (UPG) and polished grain (PG). (B) Fe and Zn concentrations in UPG and PG. EN1-EN4, four pGluB1-NAS1 independent transgenic lines. Asterisks indicate significant differences in NA, Fe or Zn concentrations between transgenic lines and WT (P≤0.05).

The NA concentrations of T_2_ rice polished and unpolished grain were determined by high-performance liquid chromatography (HPLC). Compared with the WT grain, the grains from four transgenic lines, EN1-EN4, accumulated substantial amounts of NA (Fig. 2A). In unpolished grain, the NA concentrations in EN1 to EN4 transgenic ranged from 41.0 to 65.0 µg g^−1^ of dry weight (DW), which were 3.3 to 5.2 times greater than that in the WT grain. A similar increase of NA was also observed in the polished grain. The NA concentrations in the transgenic polished grain were 23.5–47.0 µg g^−1^ DW, which were 4.1 to 8.2 times greater when compared with the WT counterparts (Fig. 2A).

### Effects of overexpression of OsNAS1 in endosperm on Fe and Zn concentrations

Because NA facilitates the intra- and intercellular transport of essential trace metal cations in plants[14], we expected that the increased NA concentration in rice would also increase the contents of Fe^2+^, Fe^3+^ and Zn^2+^. As shown in Fig. 2B, Fe concentrations in unpolished grain of transgenic lines EN1 to EN4 ranged from 15.24 and 18.68 mg kg^−1^ dry weight, respectively, which were 18.8% to 45.6% higher than that in WT (Fig. 2B). Similarly, Zn concentrations in the unpolished EN1 to EN4 grain ranged from 31.74 to 36.99 mg kg^−1^ dry weight, respectively, which were 33.4% to 55.4% higher than the WT counterparts. Zn concentrations in polished EN1 to EN4 grain ranged from 27.05 to 29.07 mg kg^−1^ dry weight, which were 22.7% to 31.9% higher than that in the WT grain. The Fe concentrations in the polished EN1 and EN2 grain were similar to that of the WT, while the Zn concentration were significantly higher than those of the WT (Fig. 2B).

### Enhancement of iron bioavailability in rice by the overexpression of OsNAS1 in endosperm

**Figure 3:**
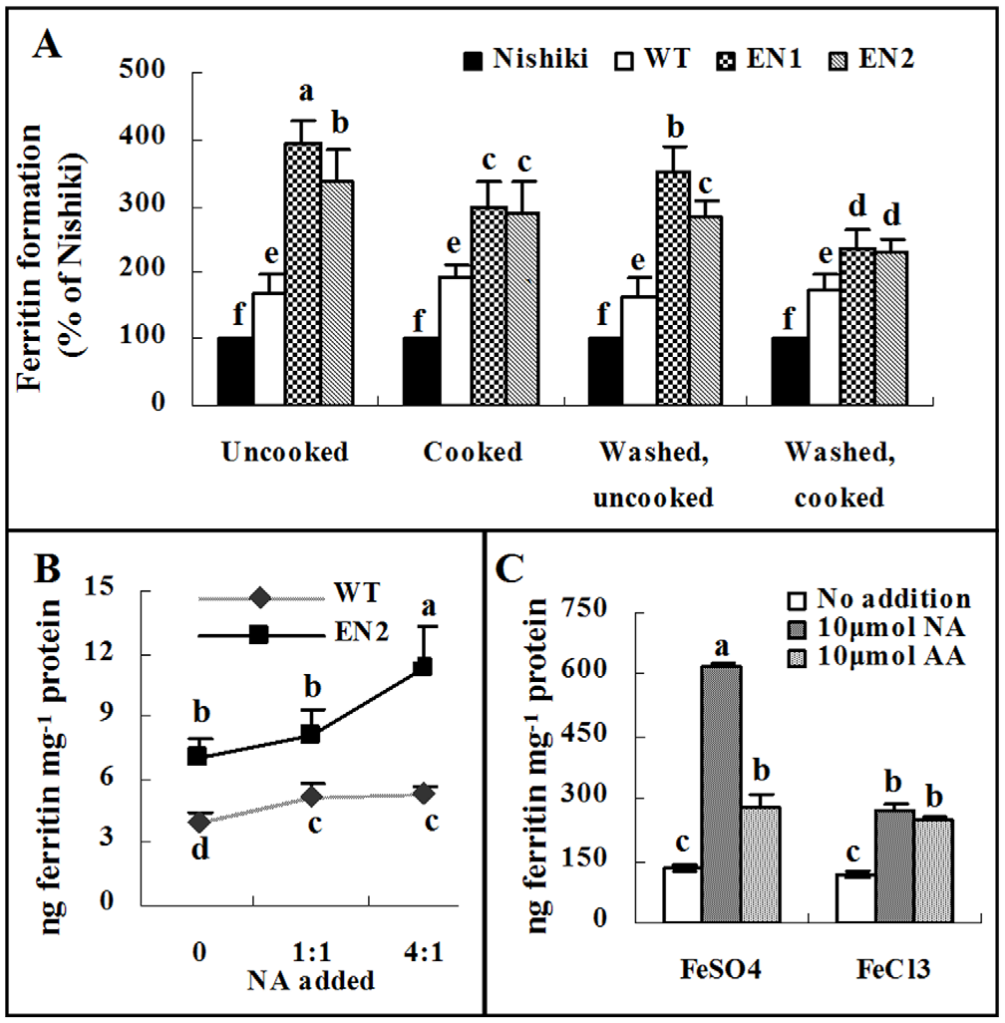
*In vitro* assessments of Fe bioavailability. (A) Formation of ferritin in Caco-2 cells treated with *in vitro* digests of untreated, cooked, washed, or washed and cooked grains of transgenic lines EN1 and EN2, and wild type (WT). Nishiki was a control rice genotype. (B) Effect of nicotianamine (NA) additions on Fe bioavailability in Caco-2 cells from transgenic and WT grain. A series of solutions containing varying molar ratios of NA:Fe (NA to Fe: 0, 1∶0, 4∶1) were used to test the effect of NA on Caco-2 cell Fe bioavailability in WT and transgenic polished rice grain. (C). Effect of the addition of NA to FeSO_4_ and FeCl_3_ solutions on Fe bioavailability to Caco-2 cells. Low case letters indicate significant differences (P≤0.05).

To assess the enhancement of bioavailable iron in the engineered rice line overexpressing *OsNAS1*, we used the Caco-2 cell line, human epithelial colorectal adenocarcinoma cells, which is widely used for *in vitro* assays to predict the absorption rate of nutrients across the intestinal epithelial cell barrier [18], [19]. After Caco-2 cells were incubated with *in vitro* digested milled rice lines (WT, EN1, and EN2), and a commonly-used standard rice variety Nishiki, ferritin concentrations in Caco-2 cells were used as a proxy for Fe bioavailability in the digests. Compared with Nishiki, Xiushi 110 WT rice grains produced greater levels (P<0.001) of ferritin (Fig. 3A). This suggests that there is variation in Fe bioavailability among different rice cultivars. Furthermore, ferritin concentrations in Caco-2 cells treated with EN1 and EN2 transgenic rice grain digests were 2.3 and 2.0 fold higher compared to the WT grains (Fig. 3A) (P<0.001).

Fe bioavailability of the washed and cooked rice grains were evaluated by washing the rice grains with Milli-Q water or cooking them at 121°C for 15 minutes prior to the *in vitro* digestion (Fig. 3A). While cooking and washing reduced the Fe bioavailability in EN1 and EN2 grains, these two transgenic lines still produced greater amounts of ferritin than the WT or the Nishiki control (Fig. 3A) (P<0.001).

### Greater potency of NA in enhancing Fe bioavailability than ascorbic acid

To explore whether the elevated NA concentrations in the transgenic grains accounted for the enhanced Fe bioavailability, synthetic NA was added to rice grain prior to the *in vitro* digestion at 1∶1 and 1∶4 molar ratios of Fe:NA. Results showed that at both ratios, the addition of NA to the WT grains enhanced (P<0.05) Fe bioavailability (Fig. 3B). Despite a higher Fe bioavailability in the transgenic grains than in the WT grains, addition of NA at 1∶1 and 4∶1 ratios to the transgenic grain further increased Fe bioavailability (Fig. 3B).

The effects of NA on ferrous sulfate (FeSO_4_) and ferric chloride (FeCl_3_) bioavailability were compared with those of AA additions. At a molar ratio of 1∶1 (Fe:enhancer), AA increased the ferrous and ferric Fe bioavailability by approximately 2 fold whereas NA enhanced ferrous and ferric Fe bioavailability by 4.7- and 2.3-fold, respectively (Fig. 3C). Notably, NA enhanced ferrous Fe bioavailability more than ferric Fe, which is in agreement with the previous observation that NA forms more stable chelates with the former than the latter oxidation states of Fe [14]. This also implies that the promoting mechanism for NA was unlikely associated with the reduction of ferric Fe to ferrous Fe by NA.

## Discussion

Polished rice is a staple food for over 50% of the world population. Because it contains little bioavailable iron for human nutrition, a large number of rice eaters are iron-deficient or anemic. Past efforts focusing on increasing only total iron concentration in edible portions of food crops have not been very effective in preventing or alleviating this widespread problem. In this study, we overexpressed a rice nicotianamine synthase gene in endosperm, and produced a significant increase in NA concentrations in rice grain. Using Caco-2 cell digest model, we have demonstrated more than two fold higher bioavailability of iron from the high NA grain than the controls. The improvement of Fe bioavailability remained after regular rice processing and cooking procedures. Our research provides a novel and potentially superior strategy to address Fe deficiency via enhancing Fe bioavailability.

**Figure 4:**
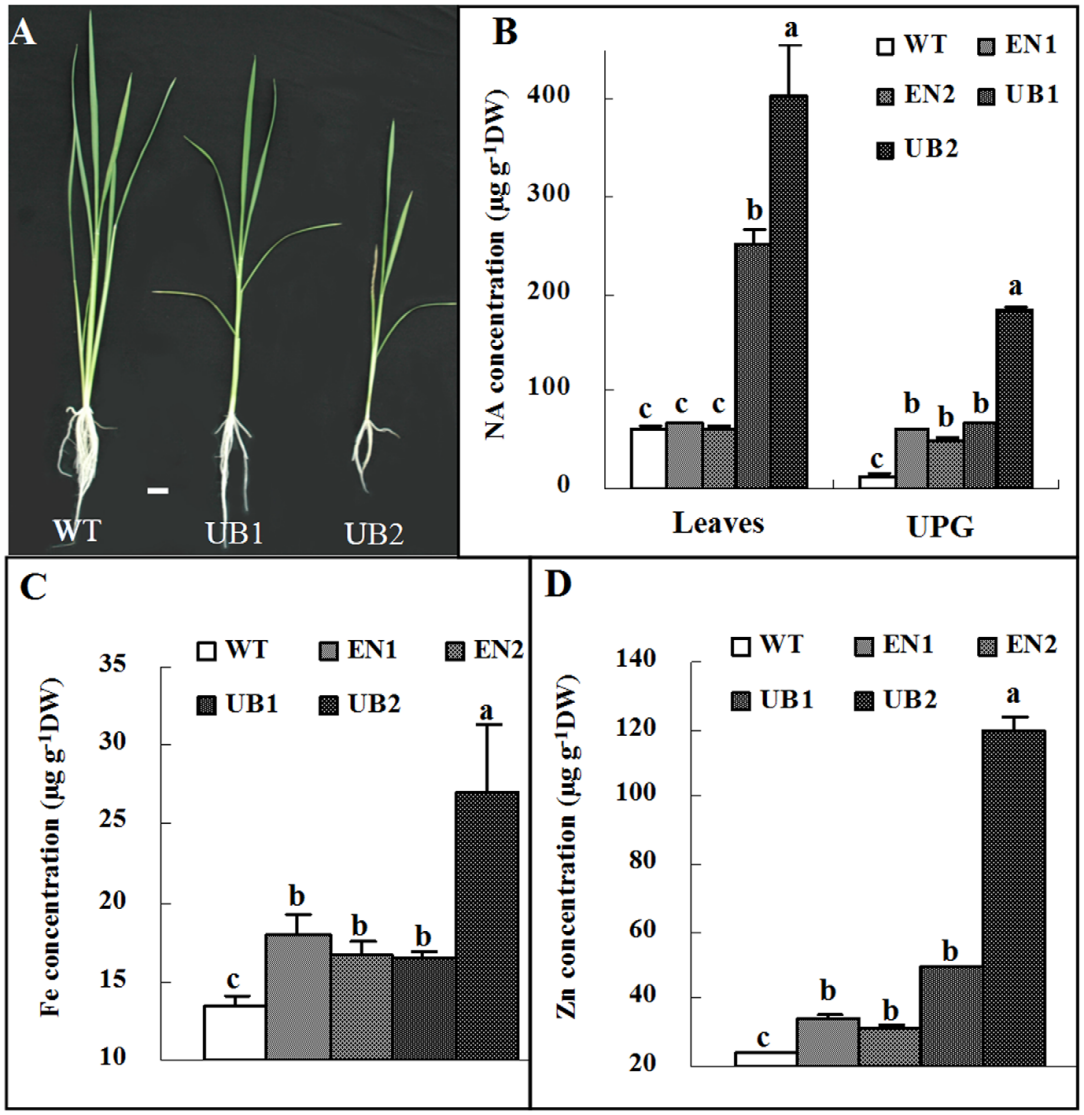
Characteristics of transgenic lines constitutively expressing NAS1 gene. (A) Thirty five day-old seedlings of UB1, UB2, and WT grown in nutrient solutions, bar = 2 cm. (B) NA concentration in leaves and unpolished grains of transgenic line EN1, EN2, UB1 and UB2. (C) and (D) Fe and Zn concentration in leaves of transgenic line EN1, EN2, UB1 and UB2, respectively. UB1 and UB2 are two transgenic lines overexpressing the *OsNAS1* gene in all tissues under the control of maize ubiquitin promoter.

NA is known as a ubiquitous metal chelator to facilitate the intercellular and intracellular transport of Fe in both Strategy I and II plant species [21], [22]. Elevated level of NA in the tobacco plants increased accumulation of Fe and other transient metal [15]. To improve Fe uptake by rice from soil, we also overexpressed *OsNAS1* in a constitutive manner by using a maize ubiquitin promoter [23]. The use of strong constitutive promoter led to a significant increase of NA contents in both leaves and grains in these transgenic plants, designed as UB1 and UB2 (Fig. 4A, 4B). Consequently, Fe and Zn concentrations in the leaves of the transgenic plants UB1 and UB2 were also significantly higher than that in the WT plants (Fig. 4C and 4D). The growth of UB1 and UB2 plants,however, was greatly inhibited (Fig. 4A). The extent of growth inhibition was correlated with the amount of NA accumulation in leaves of the plants. In contrast, endosperm specific expression of *OsNAS1* gene (EN1-4) resulted in NA accumulation in grains but not in shoots (Fig. 2A), which avoided the negative effect on agronomic performance. It would be valuable to know whether the growth inhibition in the UB1 and UB2 lines is a result of disturbed iron homeostasis, such as toxicity of overaccumulation of Fe (Fig. 4C) or Zn (Fig. 4D).

Overexpression of *OsNAS1* in our transgenic line increased Fe and Zn concentration in unpolished grains (Fig. 2B). However, in polished rice grain, only Zn content was significantly increased. Previous report showed a primary accumulation of Fe in embryo and aleurone layer of rice grain [24], which is readily lost during the polishing process. A recent study indicated that activation of *OsNAS3* by activation tagging approach (*NAS3-D* lines) increased both Fe and Zn concentration in shoots, roots and seeds, including in polished rice [16]. The discrepant effect of the overexpression of *NAS* between our data and the NAS3-D study [16] might be attributed to different expression levels of *NAS* gene achieved by using different strategies of the activation of NAS genes. It was proposed that the increase of iron concentration in the *OsNAS3* activation-tagged mutant lines resulted in the recovery of anemia in mice when the grains were used for mice feeding experiment [16]. That study, however, did not test whether the improvement in hemoglobin repletion in the experimental mice fed the OsNAS3–activation tagged grains was due to an enhanced Fe absorption by NA per se or caused by elevated amount of Fe content[16]. Our study showed that the similar amount of Fe from the high-NA rice grain EN1 and EN2 could be absorbed by the Caco-2 cells with higher efficiency than that from the WT grain (Fig. 3A). Thus, the effect of feeding *OsNAS3*-activation rice grain on the improvement of mice anemia should be the result of both increased Fe content as shown [16] and increased Fe bioavailability.

Ascorbic acid (AA) is a well known promoter of non-heme Fe absorption from plant food-based diets because of its ferric reducing and ferrous complexing properties[25]. In this study, we have demonstrated that NA exhibited an even greater enhancing effect on Fe bioavailability than AA in Caco-2 cells. Our results suggest that NA is a novel and potent promoter of Fe bioavailability that may potentially be used as a biofortifying target compound in staples and in food fortificants to improve Fe utilization by resource-poor people in the developing world who eat rice-based diets. Ongoing research is being undertaken to determine the optimal amounts of NA needed for improving Fe bioavailability.

NA was previously shown to be a strong inhibitor to angiotensin I-converting enzyme (ACE)[26], [27]. A recent study ectopically expressed a barley *NAS1* gene in rice endosperm for the purpose of production of an antihypertensive staple food [28]. Using the rice *NAS1* gene, our transgenic rice accumulated similar amount of NA in the grain. Our data showed that besides preventing hypertension, the NA over-accumulated transgenic rice has great potential in combating global human Fe deficiency in people dependent on rice for their sustenance.

## Materials and Methods

### Plant materials

The japonica variety Xiushui 110 was used for the rice transformations. Seeds were germinated in tap water for 2 days and transferred into a Yoshida nutrient solution[29] before RT-PCR analysis and phenotypic evaluation of the transgenic and WT plants.

Seeds used for elemental analyses, NA determinations and Caco-2 assays were harvested from a paddy field on the farm of the Huajia campus, Zhejiang University, with a planting distance of 18×18 cm. The soil contained 10.92 g kg^−1^ total Fe and 87.8 mg kg^−1^ total Zn as determined by inductively coupled argon-plasma mass spectrometry (ICP-MS, Agilent 7500ce, CA, USA).

### Vector construction and rice transformation

Full length *OsNAS1* ORF was amplified by RT-PCR and inserted into the binary vector pTF101.1 [30] under the control of the rice GluB-1 promoter[20] and maize ubiquitin promoter [23]. Besides the transgene expression cassette, the T-DNA region of the binary vector contained a bar gene as a selectable marker for herbicide bialaphos resistance (Fig. 1A). The resultant transformed plasmids were used for *Agrobacterium*-mediated rice transformation as described previously [31].

### RT-PCR analysis

Total RNAs were extracted from immature seeds 18 days after pollination using the TRIzol Reagent (Invitrogen, CA, USA) according to manufacturer's recommendations. The first-strand cDNA was synthesized using SuperScript II reverse transcriptase (Invitrogen, CA, USA). Semi-quantitative RT-PCR was performed using the primer pairs for *OsNAS1* and housekeeping gene *OsACTIN* (Applied Biosystems, CA, USA). The gene expression levels were determined by comparing densities of bands after agarose gel electrophoresis of the PCR products.

### Measurement of metal concentrations

Grain harvested from the field experiment was husked to obtain the unpolished grain. Portions of the unpolished grain samples were processed with a rice milling machine JNMJ3 (Taizhou Grain Instrument, Zhejiang, China) for 1 min, 3 times, to obtain polished grain. Grain samples were then ground to fine powder and digested with ultra-pure HNO_3_ and H_2_O_2_ in Teflon-coated microwave vessels. Metal concentrations were determined via ICP-MS. Each measurement was repeated three times.

### HPLC analysis of NA in rice grain and leaf

Nicotianamine assays were carried out as described below using, with minor modifications, the HPLC method reported by Wada et al[32] or using a zwitterionic hydrophilic interaction liquid chromatography (ZIC-HILIC)[33] to separate and quantify NA. Finely ground plant samples (0.5 g DW) were shaken in 10 mL of deionized water at 80°C for 2 h and centrifuged to obtain clear supernatant. A 10 µL aliquot of supernatant was combined with 10 µL of 50 mM EDTA, 20 µL 1 M borate buffer (pH 8) and 40 µL of 25 mM FMOC-Cl in acetonitrile and reacted for 30 min at 30°C to form the NA-FMOC derivative. A 10 µL aliquot of the FMOC reaction product was injected onto a Waters Symmetry C18 column (4.6×250 mm; at 40°C) with Waters Symmetry C18 cartridge guard column and chromatographed using a Dionex GS 50 gradient pump (flow rate  = 1.0 mL min^−1^) and Dionex RS 2000 fluorescence detector (excitation wavelength, 266 nm; emission wavelength, 305 nm). Elution was performed using a linear gradient of AccQ*Tag (Waters Corp., Milford, MA, USA) reagent elution solution (100%, eluent A) and acetonitrile (50% ACN in water, eluent B). The linear gradient was: 0–2 min, 80% A and 20% B; 2–20 min, 60% A and 40% B; 20–25 min, 5% A and 95% B; 25–35 min, 0% A and 100%B. Authentic nicotianamine (T. Hasegawa Co., LTD, Kawasaki-Shi, Japan) was used to construct standard curves to determine nicotianamine concentrations in the samples. Each measurement was repeated three times.

### Caco-2 cell *in vitro* digestion

Prior to *in vitro* digestion, polished rice grains were processed as follows: no treatment, cooking, washing, or washing plus cooking. For the cooking treatments, rice grains were cooked at 121°C for 15 min. For the washing treatment, 1 gram of rice grains was washed with 2 ml sterile Milli-Q water (Mill Q, Bedford, MA, USA). The samples were frozen, and then lyophilized to dryness, ground, and stored in an airtight plastic container at room temperature. One gram of freeze-dried samples was used for Fe bioavailability measurements.

The *in vitro* digestion/Caco-2 cell culture model assay was carried out to assess the Fe bioavailability in rice as described[18], [19], [34]. Cellular ferritin and total protein concentration in Caco-2 cells exposed to the rice digests were determined on an aliquot of the cell suspension with an immunoradiometric assay (RAMCO Lab, TX, USA) and a spectrophotometric assay (Bio-Rad, CA, USA), respectively. Caco-2 cells synthesize ferritin in response to increases in intracellular Fe concentration[19], hence cellular ferritin formation, expressed as ng ferritin mg^−1^ protein, was used as an index of Fe bioavailability. Experiments were conducted using six-well plates with a control sample. Polished grain from rice variety of Nishiki was used as the control sample. Fe bioavailability of each rice sample was assessed with and without NA as described. NA was added at the start of the *in vitro* digestion process. The Caco-2 experiments were replicated six times.

### Statistical analysis

Statistical significances were analyzed using SAS program (SAS Institute Inc. Cary, NC).

## References

[1] Kennedy G, Nantel G, Shetty P (2003). The scourge of “hidden hunger”: global dimensions of micronutrient deficiencies.. Food Nutr Agric.

[2] Mayer JE, Pfeiffer WH, Beyer P (2008). Biofortified crops to alleviate micronutrient malnutrition.. Current Opinion in Plant Biology.

[3] Zimmermann MB, Hurrell RF (2007). Nutritional iron deficiency.. Lancet.

[4] Welch RM (2002). Breeding strategies for biofortified staple plant foods to reduce micronutrient malnutrition globally.. J Nutr.

[5] Hazell T, Johnson IT (1987). In vitro estimation of iron availability from a range of plant foods: influence of phytate, ascorbate and citrate.. Brit J Nutr.

[6] Lynch SR, Stoltzfus RJ (2003). Iron and ascorbic acid: proposed fortification levels and recommended iron compounds.. J Nutr.

[7] Cook JD, Monsen ER (1977). Vitamin C, the common cold and iron absorption.. Am J Clin Nutr 38.

[8] Candela E, Camacho MV, Martinez-Torres C, Perdomo J, Mazzarri G (1984). Iron absorption by humans and swine from Fe(III)-EDTA. Further studies.. J Nutr.

[9] Goto F, Yoshihara T, Shigemoto N, Toki S, Takaiwa F (1999). Iron fortification of rice seed by the soybean ferritin gene.. Nat Biotechnol.

[10] Poletti S, Gruissem W, Sautter C (2004). The nutritional fortification of cereals.. Curr Opin Biotechnol.

[11] Qu LQ, Yoshihara T, Ooyama A, Goto F, Takaiwa F (2005). Iron accumulation does not parallel the high expression level of ferritin in transgenic rice seeds.. Planta.

[12] Higuchi K, Watanabe S, Takahashi M, Kawasaki S, Nakanishi H (2001). Nicotianamine synthase gene expression differs in barley and rice under Fe-deficient conditions.. Plant J.

[13] Shojima S, Nishizawa NK, Fushiya S, Nozoe S, Kumashiro T (1989). Biosynthesis of nicotianamine in the suspension-cultured cells of tobacco (*Nicotiana megalosiphon*).. BioMetals.

[14] von Wiren N, Klair S, Bansal S, Briat JF, Khodr H (1999). Nicotianamine chelates both FeIII and FeII. Implications for metal transport in plants.. Plant Physiol.

[15] Douchkov D, Gryczka C, Stephan UW, Hell R, Umlein H (2005). Ectopic expression of nicotianamine synthase genes results in improved iron accumulation and increased nickel tolerance in transgenic tobacco.. Plant, Cell & Envir.

[16] Lee S, Jeon US, Lee SJ, Kim Y-K, Persson DP (2009). Iron fortification of rice seeds through activation of the nicotianamine synthase gene.. Proceedings of the National Academy of Sciences.

[17] Cheng L, Wang F, Shou H, Huang F, Zheng L (2007). Mutation in nicotianamine aminotransferase stimulated the Fe(II) acquisition system and led to iron accumulation in rice.. Plant Physiol.

[18] Glahn RP, Cheng Z, Welch RM, Gregorio GB (2002). Comparison of iron bioavailability from 15 rice genotypes: Studies using an in vitro digestion/Caco-2 cell culture model.. J Agric Food Chem.

[19] Glahn RP, Lee OA, Yeung A, Goldman MI, Miller DD (1998). Caco-2 cell ferritin formation predicts nonradiolabeled food iron availability in an In vitro digestion/Caco-2 cell culture model.. J Nutr.

[20] Qu LQ, Takaiwa F (2004). Evaluation of tissue specificity and expression strength of rice seed component gene promoters in transgenic rice.. Plant Biotechnol J.

[21] Pich A, Scholz G, Stephan U (1994). Iron-dependent changes of heavy metals, nicotianamine, and citrate in different plant organs and in the xylem exudate of two tomato genotypes. Nicotianamine as possible copper translocator.. Plant Soil.

[22] Higuchi K, Nishizawa N, Romheld V, Marschner H, Mori S (1996). Absense of nicotianamine synthase activity in the tomato mutant ‘chloronerva’.. J Plant Nutr.

[23] Christensen AH, Sharrock RA, Quail PH (1992). Maize polyubiquitin genes: structure, thermal perturbation of expression and transcript splicing, and promoter activity following transfer to protoplasts by electroporation.. Plant Mol Biol.

[24] Tanaka K, Yoshida T, Kasai Z (1974). Distribution of mineral elements in the outer layer of rice and wheat grains, using electron microprobe X-ray analysis.. Soil Sci Plant Nutr.

[25] Hurrell RF, Lynch S, Bothwell T, Cori H, Glahn R (2004). Enhancing the absorption of fortification iron.. Int J Vitam Nutr Res.

[26] Hayashi A, Kimoto K (2007). Nicotianamine preferentially inhibits Angiotensin I-converting enzyme.. J Nutr Sci Vitaminol.

[27] Kinoshita E, Yamakoshi J, Kikuchi M (1993). Purification and identification of an angiotensin I-converting enzyme inhibitor from soy sauce.. Biosci Biotechnol Biochem.

[28] Usuda K, Wada Y, Ishimaru Y, Kobayashi T, Takahashi M (2008). Genetically engineered rice containing larger amounts of nicotianamine to enhance the antihypertensive effect.. Plant Biotechnol J.

[29] Yoshida S, Forno DA, Cock JH, Gomez KA (1976). Laboratory manual for physiological studies of rice, Ed 3..

[30] Paz M, Shou H, Guo Z, Zhang Z, Banerjee A (2004). Assessment of conditions affecting *Agrobacterium*-mediated soybean transformation using the cotyledonary node explant.. Euphytica.

[31] Chen S, Jin W, Wang M, Zhang F, Zhou J (2003). Distribution and characterization of over 1000 T-DNA tags in rice genome.. Plant J.

[32] Wada Y, Yamaguchi I, Takahashi M, Nakanishi H, Mori S (2007). Highly sensitive quantitative analysis of nicotianamine using LC/ESI-TOF-MS with an internal standard.. Biosci Biotechnol Biochem.

[33] Xuan Y, Scheuermann EB, Meda AR, Hayen H, von Wiren N (2006). Separation and identification of phytosiderophores and their metal complexes in plants by zwitterionic hydrophilic interaction liquid chromatography coupled to electrospray ionization mass spectrometry.. Journal of Chromatography A.

[34] Glahn RP, Lai C, Hsu J, Thompson JF, Guo M (1998). Decreased Citrate Improves Iron Availability from Infant Formula: Application of an In Vitro Digestion/Caco-2 Cell Culture Model.. J Nntr.

